# Evaluating Effects of Glatiramer Acetate Treatment on Amyloid Deposition and Tau Phosphorylation in the 3xTg Mouse Model of Alzheimer’s Disease

**DOI:** 10.3389/fnins.2021.758677

**Published:** 2021-10-22

**Authors:** Dawling A. Dionisio-Santos, Berke Karaahmet, Elizabeth K. Belcher, Laura D. Owlett, Lee A. Trojanczyk, John A. Olschowka, M. Kerry O’Banion

**Affiliations:** Department of Neuroscience, School of Medicine and Dentistry, Del Monte Neuroscience Institute, University of Rochester, Rochester, NY, United States

**Keywords:** Alzheimer’s disease, beta amyloid, tau phosphorylation, neuroinflammation, microglia, glatiramer acetate

## Abstract

Neuroinflammation driven by the accumulation of amyloid β (Aβ) can lead to neurofibrillary tangle formation in Alzheimer’s Disease (AD). To test the hypothesis that an anti-inflammatory immunomodulatory agent might have beneficial effects on amyloid and tau pathology, as well as microglial phenotype, we evaluated glatiramer acetate (GA), a multiple sclerosis drug thought to bias type 2 helper T (T_h_2) cell responses and alternatively activate myeloid cells. We administered weekly subcutaneous injections of GA or PBS to 15-month-old 3xTg AD mice, which develop both amyloid and tau pathology, for a period of 8 weeks. We found that subcutaneous administration of GA improved behavioral performance in novel object recognition and decreased Aβ plaque in the 3xTg AD mice. Changes in tau phosphorylation were mixed with specific changes in phosphoepitopes seen in immunohistochemistry but not observed in western blot. In addition, we found that there was a trend toward increased microglia complexity in 3xTg mice treated with GA, suggesting a shift toward homeostasis. These findings correlated with subtle changes in the microglial transcriptome, in which the most striking difference was the upregulation of *Dcstamp*. Lastly, we found no evidence of changes in proportions of major helper T cell (T_h_) subtypes in the periphery. Overall, our study provides further evidence for the benefits of immunomodulatory therapies that alter the adaptive immune system with the goal of modifying microglia responses for the treatment of Alzheimer’s Disease.

## Introduction

Recently, it has been recognized that peripheral innate and adaptive immune cells play important roles in influencing brain immunity (Brioschi and Colonna, 2019). As the resident immune cells of the central nervous system, microglia actively respond to AD neuropathology. In addition, microglia activity can be modulated by changes in peripheral inflammation (MacPherson et al., 2017). Studies have shown that patients with systemic infection and chronic inflammatory conditions are at a higher risk of developing AD (Holmes et al., 2009; Huang et al., 2015). Moreover, various systemic pro-inflammatory insults, such as peripheral LPS injections or induction of arthritis, can influence the magnitude of amyloid and tau pathology in mouse models (Lee et al., 2010; Kyrkanides et al., 2011).

One peripheral immunomodulator that has garnered attention in the search for AD therapeutics is the drug glatiramer acetate (GA, Copaxone^®^). GA is a random mixture of polymers composed of glutamic acid, alanine, tyrosine, and lysine that is currently approved for the treatment of relapsing-remitting multiple sclerosis (MS). The mechanism of GA action in mouse models of MS is unclear, but some of the major hypotheses include polarizing helper T cells to a T_h_2 phenotype through bystander effect and inducing an alternatively-activated phenotype in peripheral monocytes, which in turn decreases the infiltration of proinflammatory immune cells to the central nervous system (Prod’homme and Zamvil, 2019). In the brain, GA has been shown to promote neuroprotection by increasing the levels of neurotrophic factors such as BDNF (Aharoni et al., 2003), IGF-1 (Skihar et al., 2009), and IGF-2 (Zhang et al., 2010) and by increasing microglial secretion of anti-inflammatory cytokines such as IL-10 (Pul et al., 2011).

Glatiramer acetate may be a potential treatment for AD. Some groups have shown that peripheral administration of GA can decrease pathology in several amyloidogenic mouse models (Butovsky et al., 2006, 2007; Baruch et al., 2015; Koronyo et al., 2015; Li et al., 2020). A suggested mechanism is that GA has an immunosuppressive effect on regulatory T cells (T_regs_) in the choroid plexus, promoting monocyte migration into Aβ plaque laden areas, which increases clearance (Baruch et al., 2015; Koronyo et al., 2015). Furthermore, GA-induced infiltration of peripheral cells into the CNS is associated with an increase in IL-10 mRNA, suggesting a decrease in neuroinflammation, which may be important for GA-mediated reductions in AD pathology. These findings are consistent with earlier reports which show induction of CD11c in microglia after GA treatment (Butovsky et al., 2006). Together, these studies suggest GA may be a potent immunomodulator in the setting of AD and may have a beneficial impact on AD pathology. However, no study to our knowledge has evaluated the impact of GA on tau and amyloid pathology concurrently.

We hypothesized long-term administration of GA would improve cognitive behavior, reduce Aβ pathology, and reduce tau phosphorylation in the 3xTg mouse model of AD. We hypothesized that these effects would be mediated through an anti-inflammatory microglial phenotype promoted by a peripheral T_h_2 response. Although we observed improved novel object recognition performance and decreased Aβ load after GA treatment, we saw mixed results with tau pathology depending on phosphorylation sites. We were also able to identify microglial morphological and transcriptomic correlates to the effect on neuropathology. Altogether, we argue that in addition to decreasing plaque, GA may also modulate tau pathology; however, the precise peripheral or local immune mechanisms remain functionally elusive in our aged 3xTg mouse model.

## Materials and Methods

### Transgenic Mice

All animal procedures were reviewed and approved by the University Committee on Animal Resources of the University of Rochester Medical Center for compliance with federal regulations prior to the initiation of the study. 3xTg AD mice express mutated human APP Swedish, MAPT P301L, and PSEN1 M146V genes under control of the Thy1.2 promoter and develop Aβ plaque deposits and intraneuronal hyperphosphorylated tau aggregates with age (Oddo et al., 2003). These mice were obtained from Frank M. LaFerla and Salvatore Oddo by Howard Federoff and have been maintained as a homozygous breeding colony at the University of Rochester. Age-matched non-transgenic mice bred continuously in a parallel colony with a similar genetic background were used as controls. Approximately 15-month-old female 3xTg AD and non-transgenic mice were used for this study. This age was selected as a time when these animals reliably begin to demonstrate advanced tau phosphorylation along with amyloid pathology (Mastrangelo and Bowers, 2008). All of our mice were retired breeders obtained from a colony maintained by Dr. Allison Elder at the University of Rochester. We chose to utilize female 3xTg AD mice for our study because it has been previously reported that females develop more aggressive pathology compared to males (Mastrangelo and Bowers, 2008).

### Glatiramer Acetate Immunization

Fifteen month-old female 3xTg AD mice received subcutaneous injections of GA (ApexBio) at 500 ng/μL in phosphate-buffered saline (PBS) or PBS alone (control group) twice during the first week of treatment and then once a week for 7 additional weeks. Each mouse received 200 μL per injection. This treatment regimen was selected based on previous studies with GA in amyloidogenic models (Butovsky et al., 2006; Baruch et al., 2015; Koronyo et al., 2015).

### Novel Object Recognition

During the habituation phase, 3xTg AD mice were allowed to explore a 31 × 31 cm box for 10 mins containing two identical objects spaced ∼15 cm apart. All objects used were ceramic doorknobs of 5–6 cm in height and ∼3 cm in width. Objects and chambers were washed thoroughly with 70% ethanol before each trial. Two hours after the habituation phase, each mouse was returned to the experimental cage containing the object to which it was previously exposed (familiar object; FO) as well as a novel object (NO). Placement of the NO was randomized for each mouse. Mice were allowed to explore familiar and novel objects during a 5 min test that was videotaped for subsequent analysis using the AnyMaze Software. Scoring of the NOR performance was based on the time spent exploring both familiar and novel objects. The behavior of the mouse was considered explorative when the animal’s head faced the object with the neck extended and vibrissae moving. Simple proximity, passing-by, or standing on the object did not count as exploration. Mice that spent less that 20 s exploring both objects were not included in the analysis. Novel object exploration was calculated as discrimination index defined by the following formula:


$$\text{Discrimination}\,\text{Index}=\frac{\text{Time}\,\text{with}\,\text{NO}-\text{Time}\,\mathrm{with}\,\text{FO}}{\text{Time}\,\text{with}\,\mathrm{NO}+\text{Time}\,\mathrm{with}\,\mathrm{FO}}$$

### Immunohistochemistry

Mice were deeply anesthetized with a mixture of ketamine (i.p., 100 mg/kg) and xylazine (i.p., 10 mg/kg), and perfused intracardially with 0.15 M phosphate buffer (PB) containing 0.5% sodium nitrite (weight/volume) and 2 IU heparin/mL. For immunohistochemistry, one hemisphere was fixed over-night in 4% paraformaldehyde (PFA), pH 7.2 in 0.15 M PB at 4°C. Brains were equilibrated in 30% sucrose in PB overnight, frozen in cold isopentane and stored at −80°C. Frozen brains were then cryosectioned into 30 μm sections on a −25°C freezing stage microtome and free-floating sections were stored in a cryoprotectant solution until assayed. For immunohistochemical protocols, sections were washed to remove cryoprotectant, incubated in 3% H_2_O_2_, washed, blocked with 10% normal goat serum and incubated in primary antibody for 48 h at 4°C. For Aβ immunostaining, sections were incubated with 70 % formic acid for 3 mins after the first wash. They were then washed and incubated with secondary antibody for 2 h at room temperature and developed with Elite ABC kit and 3,3-diaminobenzidine (Vector laboratories). Sections were mounted, cleared and cover slipped in DPX (VWR). For fluorescent immunostaining, sections were incubated with secondary antibodies bound to Alexa/Dylight fluorophores (Invitrogen) and cover slipped in Prolong Gold (Invitrogen). For biotinylated primary antibodies the secondary antibody incubation was omitted, and an Alexa fluorophore conjugated to streptavidin was used. Primary and secondary antibody dilutions were as follows: biotinylated 6E10 (Invitrogen) 1:2,000; Iba1 (Wako) 1:3,000; biotinylated HT7 (Thermo Scientific) 1:10; anti-pT205 (Invitrogen) 1:5,000; anti-PHF1 (Dr. Peter Davies) 1:100; biotinylated goat anti-rabbit IgG and anti-mouse IgG (Vector) 1:2,000; goat anti-rat IgG Alexa 488 (Invitrogen) 1:1,000; goat anti-rabbit IgG Alexa 594 (Invitrogen) 1:1,000.

### Image Acquisition and Analysis

Photomicrographic images were captured by a Zeiss Axioplan Iii microscope equipped with a Sensicam (Cooke Corporations), using Slidebook 6.0 software (Intelligent Imaging Innovations, Inc.). For 6E10 histological analysis, 8-Bit grayscale images from the hippocampus were captured using a 5x objective. Subiculum boundaries were defined in ImageJ^1^ and area fractions were determined using a threshold to minimize artifact. Phospho-Tau and total tau-stained sections were imaged using a 10x objective. CA1 boundaries were defined in ImageJ, after which background was subtracted. Area fractions were then determined using an average threshold to minimize artifact.

Confocal images were obtained using an Olympus FV1000 laser scanning confocal microscope (Center Valley, PA, United States) in the Confocal and Conventional Microscopy Core of the University of Rochester Medical Center Core Facility Program. All images were acquired using sequential scanning and oversaturation was prevented by using the hi-lo feature of the FV1000 software. UPLAN objectives were used to acquire the images. For quantitative analysis, hippocampal sections were co-stained Iba1 and DAPI and imaged with a 40x oil-objective.

For microglia Sholl analysis, confocal acquired z-stacks were imported into ImageJ and compressed into a max z-projection in which individual microglia whose process arbor was within the image (8 microglia per image) were selected and cropped into a new, blank image. Images of individual microglia were thresholded to create binarized arbor outlines, despeckled once to remove artifacts, and analyzed using the semi-automated ImageJ Sholl plugin 5,53.

### ELISA and Western Blots

Hippocampi were quickly dissected, frozen in isopentane and stored at −80°C until further processing. Hippocampi were homogenized in Tissue Protein Extraction Reagent (T-PER; Thermo Scientific) at a concentration 50 mg/mL with protease and phosphatase inhibitor tablets (Thermo Scientific), vortexed and sonicated. Lysates were centrifuged at 100,000 g for 1 h. The supernatant was carefully collected and stored at −80°C. This was analyzed as the soluble fraction, bearing both monomeric and oligomeric forms of Aβ. The pellet, bearing insoluble, fibrillar Aβ, was extracted in guanidinium-HCl pH 6.0 (150 mg/mL) and centrifuged at 100,000 g for 1 h. The supernatant was stored in −80°C to be analyzed as the insoluble fraction. Soluble samples were diluted 1:5 in kit buffer for ELISA (Invitrogen). Insoluble samples were diluted 1:1,000 in kit buffer. All dilutions were established empirically.

For Western blot, hippocampal lysates were diluted 1:5 and protein concentration were determined by a BCA assay (Thermo Scientific). Protein (15 μg/lane for most blots) was electrophoresed on a Tris–HCL polyacrylamide gel and transferred to a nitrocellulose membrane (Bio-Rad) for 60 mins at 4°C. After 1 h in Western blocking reagent (Roche Diagnostics), membranes were incubated overnight with primary antibodies. After rinsing, blots were incubated with peroxidase-linked secondary antibodies (Thermo Scientific), treated with the ECL substrate (Supersignal WestDura Kit) and bands were visualized using the Azure c600 imaging systems (Azure Biosystems). Primary antibodies were: HT7 (Dako) 1:1,000; pT205 (Invitrogen) 1:1,000; and PHF1 (gift of Dr. Peter Davies) 1:1,000.

### Microglia Isolation and RNA Analysis

#### Microglia Isolation

Mice were perfused as described in section “Immunochemistry.” Brains were removed, hippocampi were dissected, and hippocampal tissue was homogenized using a Dounce homogenizer. Myelin was removed by magnetic separation using myelin depletion beads and LS columns (Miltenyi Biotec) according to the manufacturer’s protocol or through a continuous 40% percoll gradient adapted from Hammond et al. (2019). In our preliminary studies, we found that both of these methods were equally effective in terms of yield and viability (data not shown). Following myelin removal, cells were washed with FACS buffer (1× PBS containing 0.05% BSA), incubated in Fc block (2.4G2, 1:100, BioLegend), and stained with CD11b-FITC (M1/70, Biolegend), CD45-APC/Cy7 (30F11, Biolegend) and P2Ry12-APC (S16007D, Biolegend). DAPI (BD) was used as our viability marker. Microglia were defined as DAPI^–^CD45^int^CD11b^+^ cells and were sorted on a FACSAria (BD) in the University of Rochester Medical Center Flow Cytometry Core facility.

#### RNA Isolation and RNAseq

Sorted cells were collected in 300 μl RLT Buffer (Qiagen) and total RNA was isolated using the RNeasy Plus Micro Kit (Qiagen). RNA concentration was determined with the NanopDrop 1000 spectrophotometer (NanoDrop) and RNA quality assessed with the Agilent Bioanalyzer 2100 (Agilent). 1 ng of total RNA was pre-amplified with the SMARTer Ultra Low Input kit v4 (Clontech) per manufacturer’s recommendations. The quantity and quality of the subsequent cDNA was determined using the Qubit Flourometer (Life Technnologies) and the Agilent Bioanalyzer 2100 (Agilent). 150 pg of cDNA was used to generate Illumina compatible sequencing libraries with the NexteraXT library preparation kit (Illumina) per manufacturer’s protocols. The amplified libraries were hybridized to the Illumina flow cell and sequenced using the NovaSeq6000 sequencer (Illumina). Single end reads of 100 nt were generated for each sample. DESeq2-1.28.1 within R-4.0.2 (Love et al., 2014) was used to perform data normalization and differential expression analysis with an adjusted *p*-value threshold of 0.05 on each set of raw expression measures. To identify biological themes from the expression data, DeSeq2 output was run through the pre-ranked module of Gene Set Enrichment Analysis (GSEA 4.0.3, Broad Institute) (Subramanian et al., 2005). The gene ranking was based on signed 1/unadjusted *p*-value. The gene set database used was c5.all. The overrepresentation test of gene sets under “Biological Processes” was run through clusterProfiler 3.16.0 (Yu et al., 2012) with Entrez IDs of up and downregulated (*p* < 0.1) genes imported from DESeq2 output (Yu et al., 2012). *P*-values were adjusted by “BH” with a cutoff of 0.05.

#### Quantitative Real-Time PCR

To validate RNAseq results, 30 ng of cDNA generated from SMARTer Ultra Low Input kit v4 (Clontech, see Microglia Isolation) was used as input. To determine relative abundance of major transcription factors of CD4 T_h_ subsets, we used RNAeasy Mini Kit (Qiagen) on CD4^+^ cells that were magnetically isolated from the spleen (see Flow cytometry). PCR reactions were carried out in a final volume of 10 μl reactions containing TaqMan Fast Advanced Master Mix (Applied Biosystems) and Applied Biosystems Taqman Kits for *Dcstamp*, *Tbx21*, *Gata3*, and *Rorc*. Samples were denatured at 95°C for 5 min, followed by 40 cycles of denaturing at 95°C for 30 s, annealing at 60°C for 30 s and extension at 72°C for 30 s. Fold changes were determined with delta-delta Ct method.

### Flow Cytometry

Spleens were harvested from 3xTg AD and non-transgenic mice and homogenized in RPMI media by passing through a 70 μm filter to yield a single cell suspension. Red blood cells were lysed with ACK buffer (Thermo Fisher). T cells were isolated from the spleen homogenate utilizing mouse CD4^+^ T cell isolation kit, mouse (Miltenyi Biotec) and LS columns according to the manufacturer’s protocol. For CD3 and CD28 co-stimulation experiment, 96-well flat-bottom plates were coated with 200 μL per well of anti-CD3 at 10 μg/mL and incubated overnight at 4°C. After a PBS wash, 1 × 10^6^ CD4^+^ splenocytes were plated per well in complete RPMI media and anti-CD28 was added (2 μg/mL) to each well. After an 18-h 37°C, 5% CO_2_, the cells were resuspended and incubated with fixable viability dye (Zombie Yellow, BioLegend). They were then blocked with anti-CD16/32 Fc Block (BD Pharmingen, 2.4G2 1:100) for 15 mins at RT, stained for extracellular markers, fixed and permeabilized (FoxP3/Transcription Factor Staining Buffer, Invitrogen) according to manufacturer’s instructions, and stained for intracellular transcription factors overnight at 4°C in the dark. Samples were washed, read with LSRII cytometer (BD Biosciences) and analyzed with appropriate FMO and single-stained controls using FCS Express 6 (De Novo Software). The following antibodies were used: CD4-APC/Cy7 (RM4-5), CD8α-PE/CF594 (53-6.7), CD25-PE/Cy7 (PC61), CCR8-FITC (SA214G2), T-bet-PE (4B10), FoxP3-APC (FJK-165, ebioscience), Gata3-BV421 (L50-823), IL7Rα-BV605 (A7R34), CXCR3-BV711 (CXCR3-173), and CD44-BV785 (IM7). Unless otherwise mentioned, all antibodies were obtained from Biolegend.

### Statistics

Observers were blinded to subject treatment prior to behavioral tests and immunohistochemistry analysis. All statistical comparisons were performed using Prism 7.0 (Graphpad Software). A *p*-value ≤ 0.05 was considered significant. Shapiro-Wilk test was used to determine normality of the data. Based on results of normality test, Student’s *t*-test or Mann Whitney test were employed when two group means were compared. Results where more than two group means were compared were analyzed with one-way or two-way Analysis of Variance (ANOVA) depending on the number of parameters. A Tukey’s multiple comparison was used to establish significance between individual groups in such instances.

## Results

### Glatiramer Acetate Injections Improve 3xTg AD Mice Cognitive Performance

**FIGURE 1:**
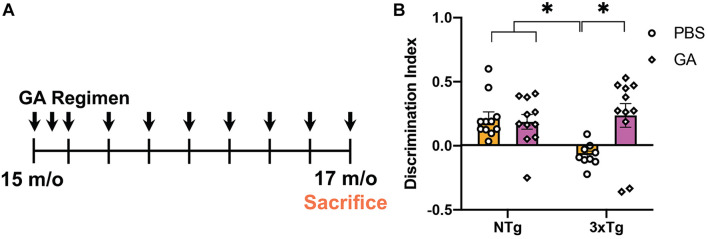
Experimental design and behavioral assessment of 3xTg AD mice after glatiramer acetate (GA) treatment. **(A)** Schematic of GA treatment regimen. 14–16-month-old 3xTg Alzheimer’s disease (AD) or non-transgenic mice received weekly subcutaneous injections with 200 μL GA at 500 ng/μL diluted in phosphate-buffered saline (PBS) for a period of 8 weeks. Mice were tested with Novel Object Recognition after 8 weeks of GA administration. **(B)** GA treated 3xTg AD mice have a significantly higher discrimination index when compared to PBS injected transgenic mice treated for 8 weeks. GA did not have a significant effect on the discrimination index of non-transgenic animals. Numerical data represented as mean discrimination index ± SEM **(B)**. *n* = 9–11 animals per group. *P < 0.05. Two-Way ANOVA with *post hoc* multiple comparisons **(B)**.

Previously, it has been shown that GA treatment can improve behavioral performance in two different amyloidogenic models (Butovsky et al., 2006; Baruch et al., 2015). We tested the cognitive abilities of aged 3xTg AD mice that received GA or PBS in subcutaneous injections for a period of 8 weeks (Figure 1A) using NOR. Some groups have reported increased anxiety in 3xTg AD mice when performing behavioral tasks that include aversive stimuli (Sterniczuk et al., 2010), so we purposely utilized this low-stress task to measure changes in cognitive impairment. Two-way ANOVA revealed that treatment with GA affected the discrimination index between the groups (Figure 1B; *F* = 4.71, DFn = 1, DFd = 38, *P* = 0.0363). Subsequent *post hoc* comparison analysis showed that PBS treated 3xTg AD mice had a significantly worse discrimination index compared to non-transgenic animals treated with PBS (*P* = 0.02) or GA (*P* = 0.04) but GA treatment significantly improved behavioral performance of 3xTg mice (*P* = 0.01).

### Glatiramer Acetate Injections Reduce Amyloid Deposits in 3xTg AD Mice

**FIGURE 2:**
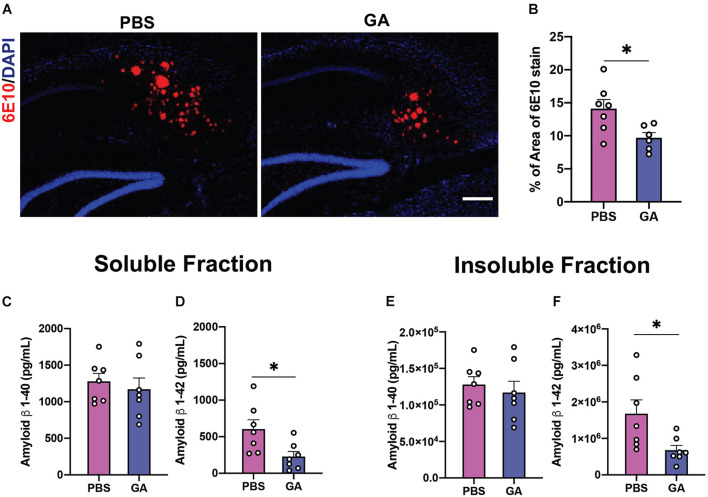
Amyloid β (Aβ) load in the hippocampus of 3xTg AD mice after GA treatment. **(A)** Representative images of Aβ plaques with 6E10 immunostaining in the subiculum of 3xTg AD mice after 8 weeks of PBS or GA injections (scale bar, 100 μm). **(B)** 3xTg AD mice injected with GA had significantly less 6E10 immunostaining. **(C–F)** ELISA for soluble and insoluble Aβ 1–40 and 1–42 demonstrated that GA-treated mice showed a significant reduction of Aβ 1–42 in both the soluble **(D)** and insoluble **(F)** fraction after the 8-week regimen. Numerical data are represented as average percent area **(B)** or pg/ml **(C–F)** of Aβ ± SEM. *n* = 6–8 animals per group. *P < 0.05. Student’s *t*-test.

Previous groups have determined that weekly treatment with glatiramer acetate reduces plaque load as well as brain tissue levels of soluble and insoluble Aβ as soon as 4 weeks (Baruch et al., 2015). We endeavored to confirm this finding in aged 3xTg AD mice. We found that weekly injections of GA for 4 weeks did not significantly reduce Aβ pathology based on immunohistochemical staining of plaque with 6E10 and concentrations of Aβ 1–40 and 1–42 (Supplementary Figure 1), which contrasts with previous findings in more aggressive amyloidogenic models. However, 8 weeks of GA treatment led to a decrease in subicular 6E10 immunostaining compared to PBS treatment (Figures 2A,B; *P* = 0.02). Eight weeks of GA treatment significantly decreased levels of hippocampal Aβ 1–42 in both the soluble (Figure 2D; *P* = 0.02) and insoluble (Figure 2F; *P* = 0.03) fractions, but not Aβ 1–40 levels (Figures 2C,E).

### The Effect of Glatiramer Acetate on Tau Phosphorylation

**FIGURE 3:**
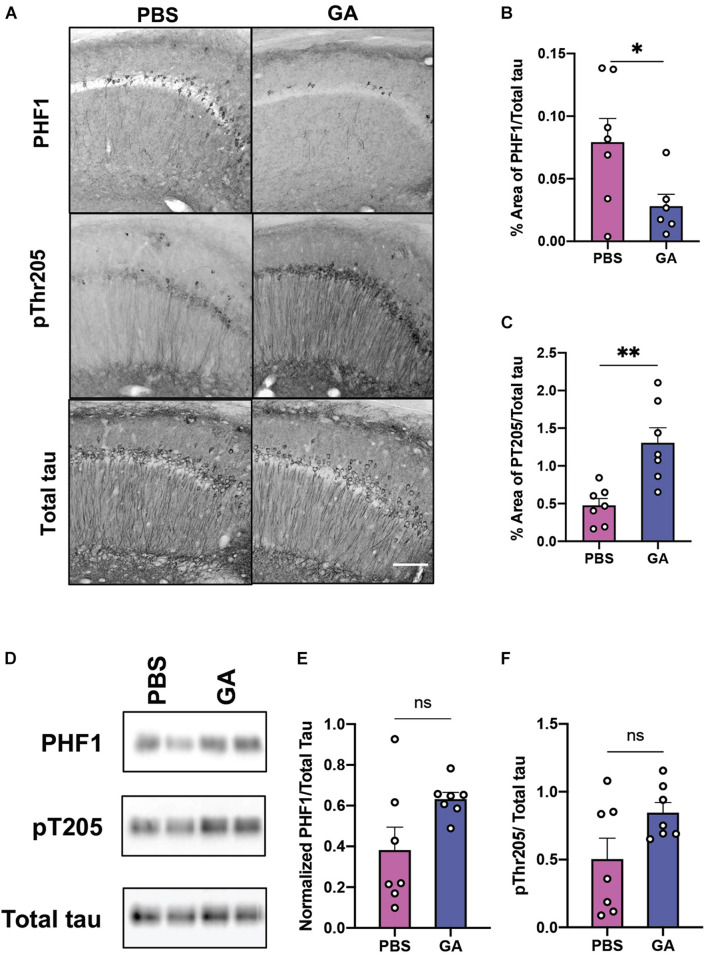
Phosphorylated tau in the hippocampus of 3xTg AD mice after GA treatment. **(A)** Representative images of tau phospho-Ser396/Ser404 (PHF1 epitope), phospho-Thr205 and total human tau immunostaining in the CA1 of 3xTg AD mice after 8 weeks of PBS or GA injections (scale bar, 50 μm). We observed a significant reduction of PHF1 staining in 3xTg AD mice injected with GA after 8 weeks of treatment **(B)**. Staining of pThr205 was significantly increased after 8 weeks of GA treatment **(C)**. Representative images of phosphorylated tau immunoblots from total hippocampal lysates after 8 weeks of PBS or GA injections **(D)**. Percent of area covered by tau stain was determined after threshold utilizing Image(J) software **(E,F)**. Numerical data are represented as area fraction phospho-tau normalized to total tau **(B,C,E,F)** ±SEM. *n* = 6–8 animals per group. *P < 0.05; **P < 0.01; ns, not significant. Student’s *t*-test or Mann-Whitney test.

Given that subcutaneous injections of GA have been reported to have anti-inflammatory and neurotrophic effects in the mouse CNS, we sought to also evaluate the effect of GA on tau pathology. We found that GA decreased PHF1 immunopositivity in the CA1 region of the hippocampus of 3xTg AD mice following 4 weeks (Supplementary Figure 1) and 8 weeks of treatment (Figures 3A,B; *P* = 0.04). The PHF1 antibody recognizes neurofibrillary tangle (NFT) phospho-epitopes on serine 396 and serine 404 that abolish the ability of tau to bind microtubules (Evans et al., 2000). In contrast, GA did not alter immunopositivity of phospho-threonine 205 (pT205) after 4 weeks (Supplementary Figure 1) and increased pT205 immunopositivity after 8 weeks of treatment (Figures 3A,C; *P* = 0.003). GA did not affect total tau stain, detected with the HT7 antibody (data not shown). Despite the changes observed with immunohistochemistry, we were not able to detect significant changes in tau phosphorylation after 4 weeks (Supplementary Figure 1) or 8 weeks of treatment utilizing western blot (Figures 3D–F).

### Glatiramer Acetate Modestly Alters Microglia Activation, Morphology and RNA Expression

**FIGURE 4:**
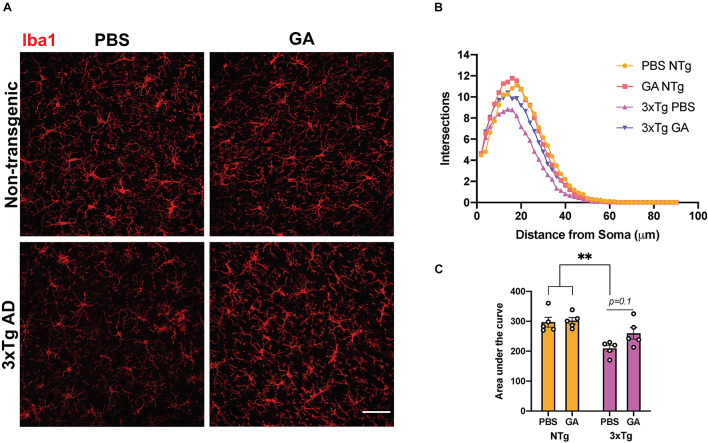
The effect of GA on microglia morphology. **(A)** Representative images show Iba1 immunopositive microglia in the hippocampus of 3xTg AD and non-transgenic mice (scale bar, 25 μm). **(B)** Sholl analysis of microglia following 8 weeks of GA treatment. **(C)** Area under the curve was used to describe the difference between the curves. Microglia from non-transgenic mice were significantly more complex than microglia from PBS injected 3xTg AD mice. Numerical data presented as mean ± SEM. *n* = 4–5 animals per group. **p < 0.01. Two-way ANOVA with *post hoc* multiple comparisons.

Given that GA is an immunomodulatory drug we decided to evaluate the phenotype and activation state of microglia. We performed Sholl analysis on Iba1-stained microglia in the hippocampus (Figures 4A,B). Curves obtained from this analysis, which summarize the distance between processes and soma, were evaluated by comparing the area under the curve (Figure 4C). Two-way ANOVA revealed that genotype significantly affected the microglia complexity between the groups (Figure 4C, *F* = 20.79, DFn = 1, DFd = 16, *P* = 0.0003). On the other hand, treatment with GA did not significantly affect microglia complexity (Figure 4C, *F* = 2.31, DFn = 1, DFd = 16, *P* = 0.1482). Post hoc comparisons revealed that PBS treated 3xTg AD mice had a significant reduction in process ramifications, a morphological state which is characteristic of activated microglia, compared to PBS (*P* = 0.0044) and GA (*P* = 0.0027) treated non-transgenic mice. GA treatment for 8 weeks did not significantly change microglia process ramifications in 3xTg AD mice when compared to PBS treated 3xTg AD mice, but the data suggest a trend toward increased microglial complexity with GA treatment (*P* = 0.1).

**FIGURE 5:**
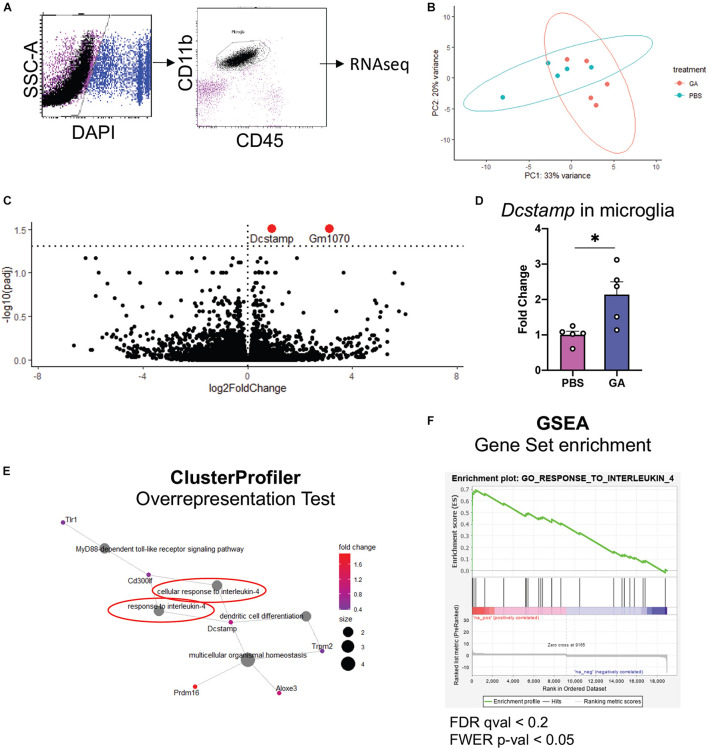
Microglial transcriptome following GA treatment. **(A)** Representative figure of workflow and FACS plots: Viable hippocampal microglia were sorted by FACS and processed for RNAseq. Data was analyzed with DESeq2. **(B)** PCA plots outlining the similarities between samples. **(C)** Volcano plot showing differentially expressed genes in green. The dashed horizontal line denotes *p* = 0.05. One statistically insignificant outlier is not displayed due to excessively high log2FoldChange. **(D)** qRT-PCR was used to confirm the upregulation of *Dcstamp*. **(E,F)** Gene ontology-based analyses reveal enrichment of pathways pertinent to IL-4 response. **(E)** Overrepresentation test was performed with the upregulated genes that had *p* < 0.1. 13 genes met these criteria. Gene-Concept Network shows enrichment of “cellular response to interleukin-4” and “response to interleukin-4” (circled in red, **E**). Immune cell activation/differentiation along with IL-4 response seems to be a common functional module. **(F)** GSEA plot showing the only significantly positively enriched term (FWER; *p* < 0.05) in microglia isolated from GA-treated animals in our pre-ranked GSEA. *P < 0.05, Student’s *t* -test.

To further assess microglial changes after 8 weeks of GA treatment, we performed RNAseq on CD11b^+^CD45^int^ cells isolated from the hippocampi of GA- and PBS-treated 3xTg AD mice (Figure 5A). We found that the only statistically significant (*P* < 0.05) differentially expressed genes were *Gm1070* and *Dcstamp* (Figure 5C), which were both upregulated in microglia from GA treated mice. We confirmed the upregulation of *Dcstamp* in RNA isolated from microglia through RT-PCR (Figure 5D, *P* = 0.02).

One limitation of RNAseq is the difficulty in obtaining biological insight from genome-wide expression data. In particular, subtle changes may be hidden by technical noise. Moreover, focusing solely on select genes that reach the statistical thresholds may mask the biological pathways involved (Subramanian et al., 2005). To extract this type of information, we performed Overrepresentation Analysis (i.e., hypergeometric test) with clusterProfiler R package on genes that showed *p* < 0.1 when comparing PBS and GA treated 3xTg mice, and Gene Set Enrichment Analysis (GSEA) on DESeq2 output. In both types of analysis, we noted a significant enrichment of pathways related to IL-4 response (Figures 5E,F). In fact, the only gene set that was significantly positively enriched (FWER; *p* < 0.05) in microglia isolated from GA-treated mice was “GO_Response_to_Interleukin_4” in GSEA analysis.

### Glatiramer Acetate Does Not Modify CD4^+^ T_h_ Cell Proportions in the Periphery

**FIGURE 6:**
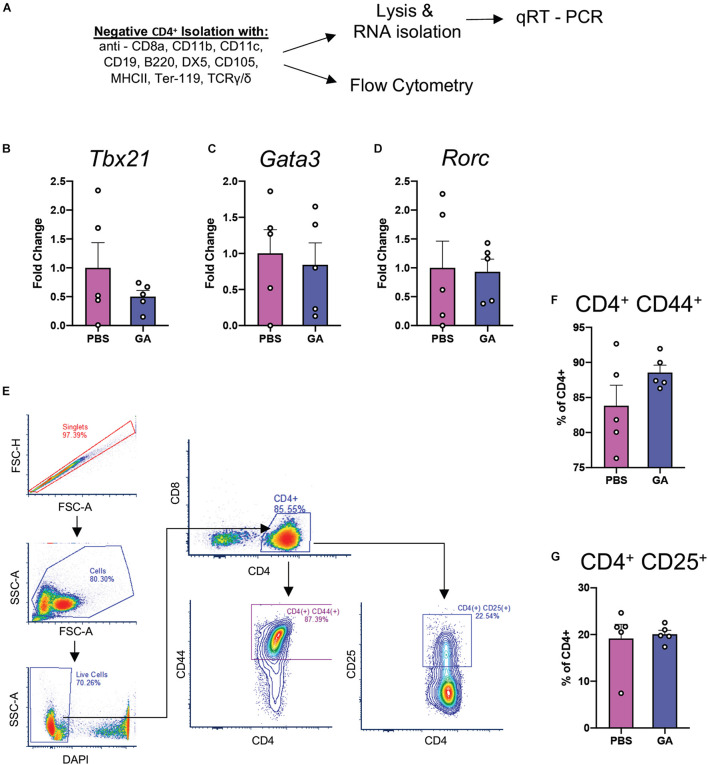
CD4^+^ T_h_ responses at 8 weeks of GA treatment. **(A)** Schematic representing isolation of splenic CD4^+^ T cells for flow cytometry and qRT-PCR analyses of major T_h_ transcription factors: *Tbx21* (T_h_1, **B**), *Gata3* (T_h_2, **C**), and *Rorc* (T_h_17, **D**). We couldn’t detect statistically significant differences between any of the comparisons. **(E)** Representative gating strategy to quantify activated T cells (CD4^+^CD44^+^) and Tregs (CD4^+^CD25^+^) in the samples isolated in panel **(A)**. We were not able to detect any statistically significant differences in CD4^+^CD44^+^ **(F)** or CD4^+^CD25^+^ **(G)** percentages. Numerical data presented as mean ± SEM. *n* = 4–5 animals per group. Student’s *t*-test.

Glatiramer acetate has been reported to induce a shift toward type 2 immune responses in the periphery (He et al., 2014). We chose to investigate peripheral CD4^+^ T cell populations in order to determine whether changes in their immunophenotype could be correlated with changes in microglia and pathology. We performed quantitative real-time PCR (qRT-PCR) experiments on isolated CD4^+^ splenocytes for the major transcription factors of T_h_1, T_h_2, and T_h_17 cells: Tbx21, Gata3, and Rorc (Figures 6A–D). We did not find a significant difference in the expression of these transcription factors in splenocytes after 8 weeks of GA treatment. Further, we attempted to identify whether percentages of splenic CD4^+^CD25^+^ cells (T_regs_) had been impacted by GA treatment through flow cytometry (Figure 6E). We did not detect any statistically significant changes in proportions of T_regs_ as well (Figure 6F). We also did not observe a statistically significant difference in percentages of CD4^+^CD44^+^ cells (Figure 6G) which are known to correspond to activated T cells.

Given that the temporal dynamics of the immune response to GA may have been a contributing factor, we chose to characterize CD4^+^ T-cell activation profiles in a different cohort of mice at a 4 week time point. To ask whether CD4^+^ T cells were primed toward a certain type of T cell activation profile, we re-stimulated splenic CD4^+^ cells from the same cohort for 18 h with CD3 and CD28 (Supplementary Figure 2A). However, we did not detect any change in their phenotype through intracellular staining with TBET, GATA3, or FOXP3 for flow cytometry (Supplementary Figures 2B–D) despite being able to confirm decreased PHF1 immunostaining in this cohort, similar to what was observed in other cohorts with 4 and 8 weeks of GA treatment (Supplementary Figure 2E).

## Discussion

In this study we present evidence of potential simultaneous benefits of glatiramer acetate on amyloid pathology and tau phosphorylation, and associated changes in microglia. GA has been used for the treatment of relapsing-remitting multiple sclerosis for more than two decades. Over the last decade, its effects have been found to be beneficial in mouse models of disorders with a prominent neuroinflammatory component such as ischemic injury, glaucoma and Huntington’s disease (Bakalash et al., 2005; Corey-Bloom et al., 2014; He et al., 2014; Reick et al., 2016; Cruz et al., 2018). In agreement with what has been previously reported in other models of neurodegeneration (Rentsendorj et al., 2018; Doustar et al., 2020), we found that GA improved cognitive performance in 3xTg AD mice. Specifically, the performance of GA-treated 3xTg AD mice in the NOR task is comparable to that of non-transgenic mice.

In previous studies using established mouse models of AD, such as 5xFAD or APP/PS1, GA reduced plaque pathology after only 4 weeks of treatment (Butovsky et al., 2006). This contrasts with our data in the 3xTg AD mouse model, which required treatment with GA for 8 weeks before amyloid plaque reduction was evident. One potential reason for this difference could be the plaque dynamics through the lifespan of 3xTg AD mice and other amyloidogenic models. For example, 5xFAD mice tend to develop aggressive pathology across the hippocampus and the cortex starting as early as 2 months of age, while Aβ plaques in our 3xTg AD mouse line are largely limited to the subiculum and start accumulating at 15 months of age (Mastrangelo and Bowers, 2008). Furthermore, microglia and other immune cells in aged mice might respond differently to GA treatment and other immunomodulatory approaches. In particular, single-cell characterization of microglia has revealed distinct transcriptional profiles between aged and young animals which could partly explain the discrepancy in response to GA between different models (Hammond et al., 2019). In contrast to the C57BL/6 background of 5xFAD and many APP/PS1 model variations, the 3xTg AD mice are on a mixed background. This may introduce variables that are beyond the scope of our study. Lastly, while we did not design these experiments to make a direct comparison between our 4- and 8-week treatment groups, there seems to be an apparent effect of GA on amyloid accumulation rather than clearance by microglia. A potential future direction for these studies would be to evaluate the effect of chronic GA treatment in young mice who have yet to develop pathology.

We saw a decrease in normalized PHF1 immunostaining, which identifies a phosphorylation on serine 396 and serine 404, after 8 weeks of treatment with GA (Figure 3B). However, we saw a significant increase in normalized pT205 immunostaining after 8 weeks of treatment that contrasted with the decrease in PHF1 (Figure 3C). While the contrast between the two different phospho-epitopes is surprising, one possible reason for this observation could be that GA is particularly effective in slowing Tau pathology characterized by PHF1. This may in turn disrupt the biochemical balance of phosphorylation events and therefore, lead to increased pT205 signal. The differential impact of GA on various phosphorylation sites on Tau should be more closely examined in future studies involving different time points and more phospho-epitopes.

Notably, we were not able to detect significant changes in phosphorylated tau in hippocampal homogenates utilizing Western blot. Such findings may be due to differences in epitope availability or other features unique to tissue processing and antigen detection between Westerns Blots and immunohistochemistry experiments; but we nevertheless acknowledge that the reasons behind the discrepancy are difficult to conceptualize. Additionally, we note substantial intragroup variability of phospho-epitope levels in Western blots. Future studies are required to provide clarification.

Our results suggest that peripheral administration of GA for 8 weeks induces a subtle change in the microglia of 3xTg AD mice. We observed a trend toward an increase in ramification of hippocampal microglia after GA treatment (Figure 4C). This morphology could point to a shift toward a homeostatic phenotype, which may contribute to neuronal health, and therefore lead to improved cognitive function which was evident in our behavioral task. In addition, we observed moderate changes in microglial RNA expression after GA treatment (Figure 5B). The only statistically significant findings were the upregulation of *Gm1070*, which is a predicted gene, and *Dcstamp* (Figure 5C). Although *Dcstamp* is a relatively unstudied gene with regard to AD pathology, its expression is upregulated in 4 month old 5xFAD mice compared to non-transgenic controls (Griciuc et al., 2019). Interestingly, it was downregulated in 5xFAD;Trem2^–/–^ mice compared to the 5xFAD in the same study, perhaps suggesting that it is a component of the transcriptional profile downstream of Trem2 (e.g., Disease-Associated Microglial or DAM). This is of particular importance since Trem2 is hypothesized to play a major role in differentiation of microglia to the DAM phenotype and helping to ameliorate amyloid pathology (Keren-Shaul et al., 2017; Ulland and Colonna, 2018; McQuade et al., 2020). Similarly, *Dcstamp* was found to be upregulated in CD11c^+^ microglia, which have been shown to interact with plaques (Butovsky et al., 2006). Therefore, GA treatment may be associated with certain aspects of DAM differentiation, but further studies are needed to establish this correlation and determine the functional significance of *Dcstamp* in AD. Future studies should also investigate if a microglial subpopulation is driving the increased Dcstamp signal or if the expression has a global pattern. While we could not get antibodies established in bone (Kukita et al., 2004) to work for DCSTAMP IHC, perhaps due to differences in tissue processing, further studies involving a reporter model are underway.

DCSTAMP, also known as TM7SF4, has been observed to play critical roles in maintaining healthy bone structure through cell–cell fusion events that lead to osteoclastogenesis and ultimately, bone resorption (Kukita et al., 2004; Yagi et al., 2005). DCSTAMP contains an Immunoreceptor Tyrosine-based Inhibitory Motif (ITIM), which is required for the aforementioned cell fusion (Chiu et al., 2012). Interestingly, dendritic cells generated from *Dcstamp*^–^*^/^*^–^ mice display increased antigen presentation activity, resulting in increased activation of antigen-specific T cells (Sawatani et al., 2008). They are also able to phagocytose better, which suggests that DCSTAMP might inhibit phagocytosis within the context of dendritic cells (Sawatani et al., 2008). In our AD model, *Dcstamp* expression and decreased plaque load correlate. While this is unexpected if DCSTAMP has similar functions across microglia and dendritic cells, we hypothesize that it might be part of a negative feedback pathway that fine tunes inflammation associated with the mechanisms that decrease plaque load. Ultimately, this may maintain a homeostatic environment in which microglia can become more ramified (Figure 4C) and clear plaque better (Figure 2B).

Although invading monocytes (CD45^hi^CD11b^+^) and increased expression of leukocyte trafficking molecules have been observed with GA treatment, using flow cytometry we observed virtually no invading monocytes in the hippocampus of 3xTg AD mice (Figure 5A). Once again, this discrepancy could relate to differences in age, background, or pathology between the 3xTg AD and 5xFAD models. We further limited our RNAseq input to microglia. Nevertheless, we cannot rule out whether invading monocytes took on a profile similar to microglia earlier during GA treatment.

Our geneset-based approaches for analyzing the microglial transcriptome demonstrated enrichment of pathways related to response to IL-4 (Figures 5E,F), which suggests an overall alternatively-activated microglial phenotype (Varin and Gordon, 2009). While we acknowledge that such categorizations are likely limited and may be misleading in explaining microglial phenotype heterogeneity, our *in silico* findings are in general agreement with previously established hypotheses that GA is an anti-inflammatory drug.

Glatiramer acetate is widely considered to modulate the adaptive immune system. We investigated the peripheral immune system to determine if there were GA-associated immunophenotypic changes in the 3xTg AD mice. We could not observe any statistically significant differences in T_h_1, T_h_2, or T_h_17 proportions after 8 weeks of GA treatment. Although this is inconsistent with some reports in the literature, GA has also been shown to impact peripheral innate immunity. For example, in previous studies, GA was found to be protective against Experimental Autoimmune Encephalomyelitis (EAE) in *Stat6*^–/–^ and *IL4*^–/–^ *IL10*^–/–^ mouse models (Weber et al., 2007). This suggests that non-T cell-based mechanisms may also be at play, since *Stat6* is a major transcription factor involved in production of T_h_2 specific cytokines. Since we did not detect activation of CD4^+^ T cells through CD44 immunopositivity, we argue that future studies, in our model, should scrutinize the impact of GA on monocytes or other innate immune cells when attempting to elucidate the mechanism by which peripheral administration impacts local pathology.

In conclusion, GA administration appears to have a beneficial effect in the 3xTg AD mouse model. We provide evidence that weekly treatment for 8 weeks improves cognitive performance and decreases Aβ pathology, while the effect on tau pathology is less clear. These changes are correlated with a subtle change in microglia morphology and RNA expression, most significantly characterized by upregulation of *Dcstamp*. In addition, this transcriptional profile seems to be positively enriched in pathways pertinent to response to IL-4, which is consistent with the established status of GA as an anti-inflammatory drug. However, these changes do not appear to correlate to immunophenotype or activation status of peripheral CD4^+^ T cells, perhaps suggesting the involvement of innate immune mechanisms. Further research is needed to establish the mechanism connecting peripheral response to GA with microglial phenotype and AD pathology in this mouse model. Similarly, the functional significance of the microglial transcriptome induced by GA warrants more research. Nonetheless, this work provides additional evidence for the use of GA in AD.

## Data Availability Statement

The datasets presented in this study can be found in online repositories. The names of the repository/repositories and accession number(s) can be found below: https://www.ncbi.nlm.nih.gov/geo/query/acc.cgi?acc=GSE169216.

## Ethics Statement

The animal study was reviewed and approved by University Committee on Animal Resources of the University of Rochester Medical Center.

## Author Contributions

DD-S, BK, and MKO designed the research. DD-S, BK, and LAT performed the research. DD-S and BK analyzed the data and wrote the manuscript. EB, LO, MKO, and JO provided input throughout the process. All authors reviewed and approved the manuscript.

## Author Disclaimer

The content is solely the responsibility of the authors and does not necessarily represent the official views of the National Institute of General Medical Science or NIH.

## Conflict of Interest

The authors declare that the research was conducted in the absence of any commercial or financial relationships that could be construed as a potential conflict of interest.

## Publisher’s Note

All claims expressed in this article are solely those of the authors and do not necessarily represent those of their affiliated organizations, or those of the publisher, the editors and the reviewers. Any product that may be evaluated in this article, or claim that may be made by its manufacturer, is not guaranteed or endorsed by the publisher.

## Footnotes

1
http://rsb.info.nih.gov/ij/
