# Supplementary material for: Evaluating Effects of Glatiramer Acetate Treatment on Amyloid Deposition and Tau Phosphorylation in the 3xTg Mouse Model of Alzheimer’s Disease
Source: Front Neurosci. 2021 Oct 22;15:758677. doi: 10.3389/fnins.2021.758677 (PMC8569891; doi:10.3389/fnins.2021.758677)
Supplement: Supplementary file 1 [file Data_Sheet_1.PDF]

Supplementary Figure 1

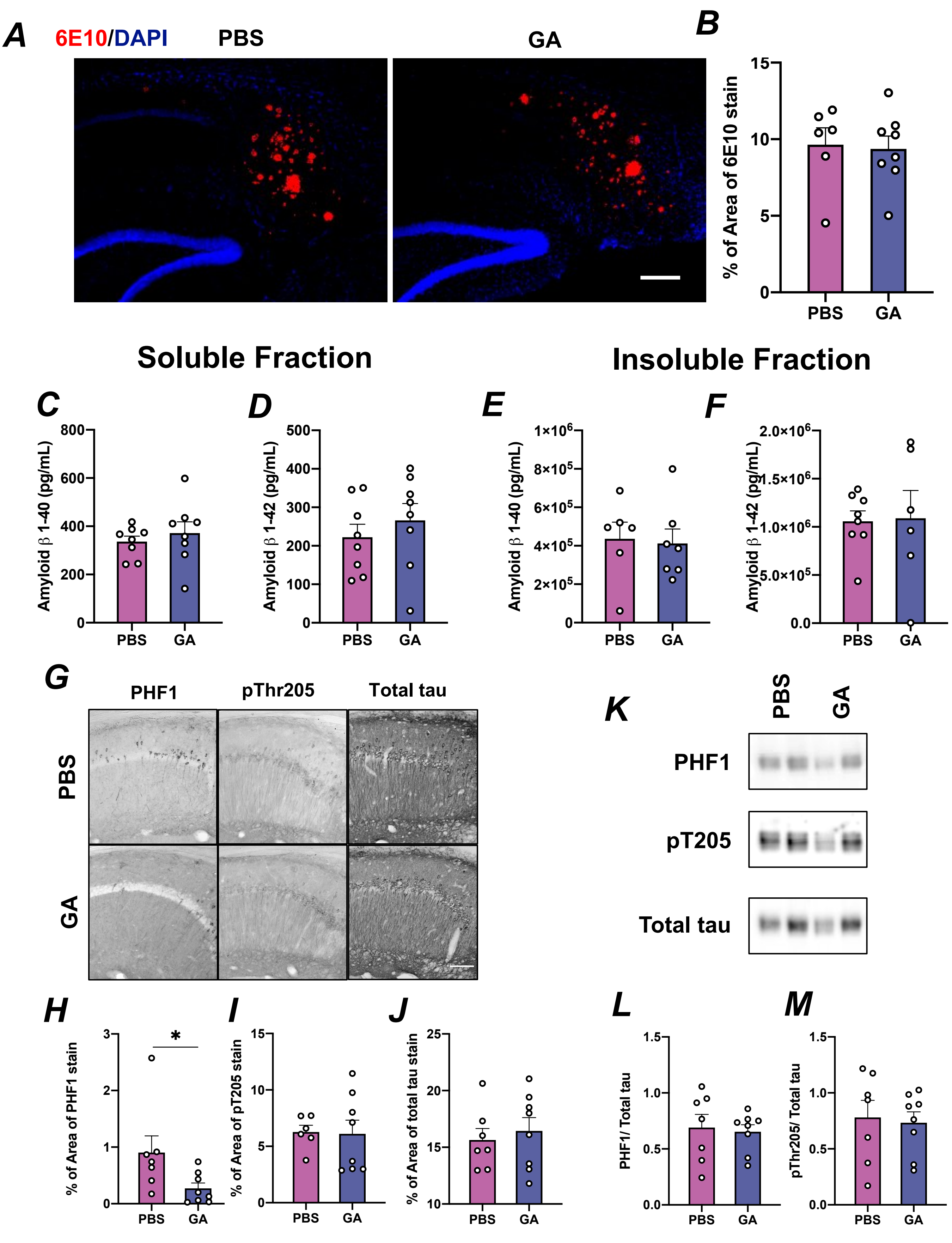

Supplementary Figure 1. Amyloid β load and phosphorylated Tau staining in the hippocampus after 4 weeks of GA treatment. (**A**) Representative images of Aβ plaques with 6E10 immunostaining in the subiculum of 3xTg AD mice after 4 weeks of PBS or GA injections (scale bar, 100 μm). (**B**) 3xTg AD injected with GA had no difference in immunostaining at this time-point. (**C-**F) ELISA for soluble and insoluble Aβ 1-40 and 1-42 demonstrated that GA-treated mice did not have a significant reduction of Aβ 1-42 in either the soluble (**C-D**) and insoluble (**E-F**) fraction after the 4-week regimen. (**G**) Representative images of PHF1 epitope, phospho-Thr205 and total human tau immunostaining in the CA1 of 3xTg AD mice after 4 weeks of PBS or GA injections (scale bar, 50 μm). We observed a significant reduction of PHF1 staining in 3xTg AD mice injected with GA after 4 weeks of treatment (**H**). Staining of pThr205 and total tau was not significantly changed after 4 weeks of GA treatment (**I, J**). Representative images of phosphorylated tau immunoblots from total hippocampal lysates after 4 weeks of PBS or GA injections (**K**). We did not observe a significant difference in phospho-tau protein levels after 4 weeks of GA (**L, M**). In all panels, numerical data represent mean ± SEM. n=6-8 animals per group. * $P<0.05$. Student's *t*-test or Mann-Whitney test.

Supplementary Figure 2

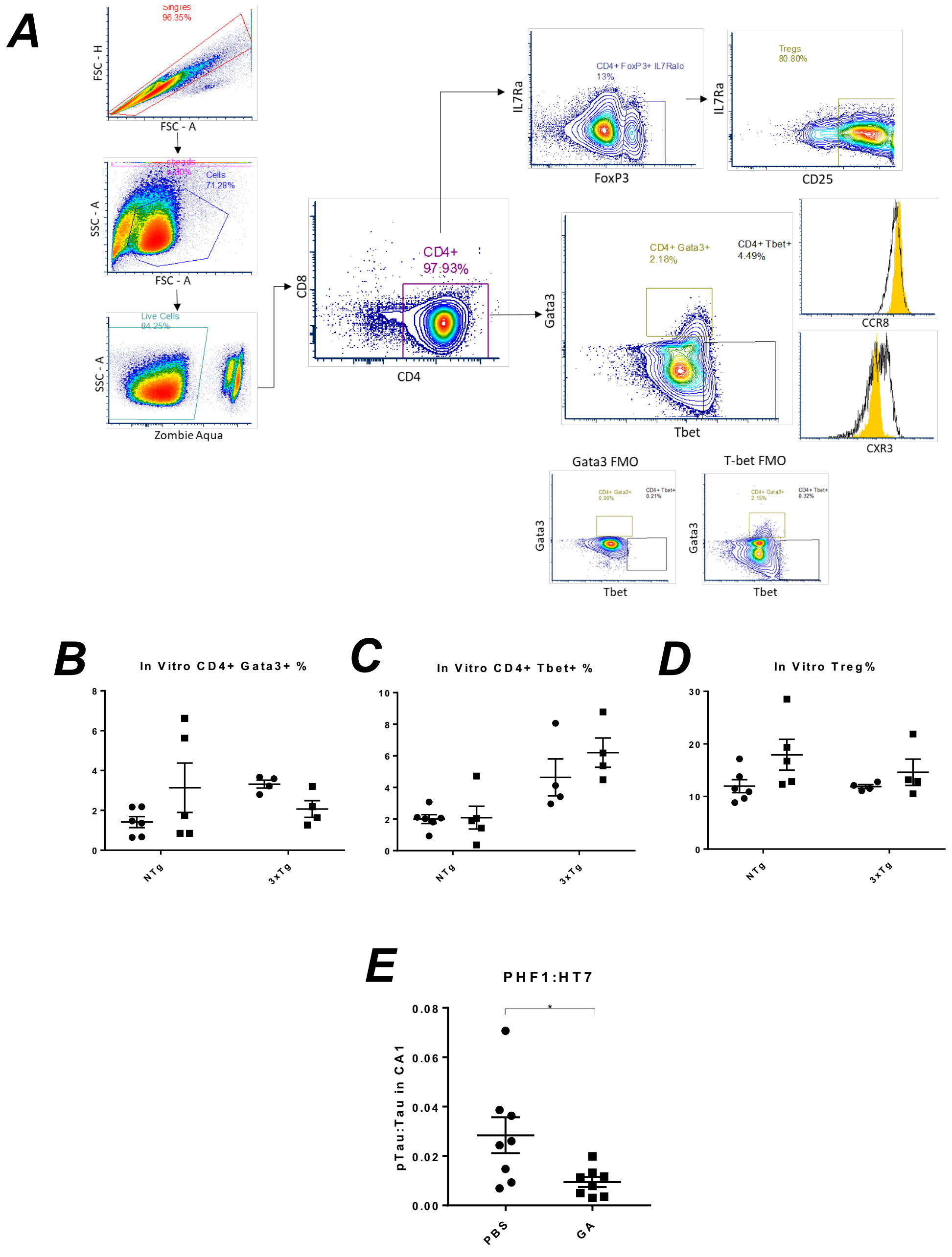

Supplementary Figure 2. Restimulation of splenic CD4$^+$ T cells following 4 months of GA treatment. (**A**) Representative image of gating strategy. In addition to FMO controls, CXCR3 and CCR8 were used to validate the gating of T_h1 and T_h2 populations. Immunophenotyping of re-stimulated splenic CD4$^+$ T cells following 4 weeks of GA treatment (**A-D**). We did not detect any bias towards a T_h subtype in isolated splenic CD4$^+$ cells following 18 hours of re-stimulation with CD3/CD28 (**B-D**). Nevertheless, we were able to confirm that GA-treated mice in this cohort had decreased PHF1 staining intensity at this time point (**E**).
